# Self-formation of concentric zones of telencephalic and ocular tissues and directional retinal ganglion cell axons

**DOI:** 10.1101/2023.03.22.533827

**Published:** 2023-03-24

**Authors:** Wei Liu, Rupendra Shrestha, Albert Lowe, Xusheng Zhang, Ludovic Spaeth

**Affiliations:** 1 Department of Ophthalmology and Visual Sciences; 2 Department of Genetics; 3 The Ruth L. and David S. Gottesman Institute for Stem Cell Biology and Regenerative Medicine; 4 Dominick P. Purpura Department of Neuroscience Albert Einstein College of Medicine, Bronx, NY 10461

**Keywords:** human pluripotent stem cell, organoid, telencephalon, optic stalk, optic disc, neuroretina, tissue patterning, self-organization, retinal ganglion cell, axon growth and pathfinding, cell surface protein, cell isolation, glaucoma

## Abstract

The telencephalon and eye in mammals are originated from adjacent fields at the anterior neural plate. Morphogenesis of these fields generates telencephalon, optic-stalk, optic-disc, and neuroretina along an axis. How these telencephalic and ocular tissues are specified coordinately to ensure directional retinal ganglion cell (RGC) axon growth is unclear. Here, we report the self-formation of human telencephalon-eye organoids comprising concentric zones of telencephalic, optic-stalk, optic-disc, and neuroretinal tissues along the center-periphery axis. Initially-differentiated RGCs grew axons towards and then along a path defined by adjacent PAX2+ optic-disc cells. Single-cell RNA sequencing identified expression signatures of two PAX2+ cell populations that mimic optic-disc and optic-stalk, respectively, mechanisms of early RGC differentiation and axon growth, and RGC-specific cell-surface protein CNTN2, leading to one-step purification of electrophysiologically-excitable RGCs. Our findings provide insight into the coordinated specification of early telencephalic and ocular tissues in humans and establish resources for studying RGC-related diseases such as glaucoma.

## Introduction

Our understanding of eye and brain development in humans is mostly deduced from animal studies. In mice, fate mapping of the anterior neural plate reveals that the eye field is in rostral regions surrounded anteriorly and laterally by the telencephalic field and caudally and medially by the hypothalamic field, indicating the proximity of their embryonic origins ^1^. Subsequent evagination of the eye field generates bilateral optic vesicles and optic stalks; the optic stalks connect the optic vesicles to the forebrain, forming a midline-periphery axis. The optic vesicle then invaginates ventrally, resulting in the formation of double-layered optic cups in which the inner and outer layers develop into the neuroretina and retina pigment epithelium (RPE), respectively. The posterior pole of the optic-cup forms the optic disc (also known as optic nerve head). In the central neuroretina close to the nascent optic disc, retinal ganglion cells (RGCs) start to appear as the optic fissure nearly closes. Early RGC axons find their path toward the optic disc and then enter the optic stalk to reach the brain. Concentrically organized growth-promoting and growth-inhibitory guidance molecules around the optic disc regulate RGC axon growth and pathfinding through multiple mechanisms ^2–4^. Coinciding with the optic-cup formation, the surface ectoderm facing the optic vesicle develops into lens. Collectively, the early development of telencephalic and ocular tissues is highly coordinated in mammals.

Early telencephalic and eye development is marked and regulated by a group of tissue-specific transcription factors and signal transduction molecules ^5^. In mice, Foxg1 is specifically expressed in the presumptive telencephalon and is essential for the development of the cerebral hemispheres ^6^. Pax6 is specifically expressed in the eye field and is essential for the development of multiple ocular lineages, such as the neuroretina, lens, and RPE ^7–10^. Vsx2 and Mitf are specifically expressed in the neuroretina and RPE, respectively, and are essential for retinal development ^11–14^. Pax2 is expressed in the optic stalk, optic vesicles, central neuroretina, and optic disc; Pax2 is essential for optic stalk and nerve development ^15–18^. Fgf8 is specifically expressed at the rostral forebrain at early stages, induces Foxg1 expression ^19^, and regulates telencephalic patterning in a dose-dependent manner ^20^. Fgf8 also maintains Pax2 expression in the optic stalk ^21^, and Fgf8 and Fgf3 coordinate the initiation of retinal differentiation in chicks ^22,23^. Despite these findings in vertebrates, it is unclear how telencephalic and ocular tissues are specified in humans.

Human organoids are transformative since they enable us to study the mechanisms of cell specification and differentiation directly in humans ^24^. Self-organized three-dimensional (3-D) retinal organoids are originally reported by Sasai’s group and further improved in follow-up studies ^25–33^. We and others have demonstrated that retinal organoids derived from human embryonic stem cells (hESCs) display a stratified structure containing all major types of retinal cells. Although RGCs are differentiated in 3-D retinal organoids, there is no proper RGC axon outgrowth toward the optic disc as seen in vivo since the optic disc-like tissue is missing in retinal organoids. When these organoids are dissociated into single cells or cut into pieces for adherent culture, RGCs generate neurites ^34–36^. A variety of brain organoids have been described ^37–41^. Although rudimentary ocular tissues are occasionally found in some brain organoids ^42^, ocular and brain tissues are not patterned along any defined axis. Assembloids, in which retinal, thalamic, and cortical organoids are assembled ^32^, do not contain any optic disc and stalk tissues. Collectively, RGC axon outgrowth and pathfinding directed by optic disc and stalk tissues in organoids have not been reported. Since RGCs degenerate in glaucoma and cell replacement therapies are actively evaluated, it is imperative to establish models for studying RGC axon growth and pathfinding in humans.

Tissue patterning and coordinated specification are fundamental for body plan formation in vivo. Remarkably, concentric zones of trophectoderm, endoderm, mesoderm, and ectoderm ^43,44^, neural plate and neural plate border ^45^, and ectodermal cells ^46^ are self-organized from single pluripotent stem cells, indicating patterning and coordinated specification of these tissues in vitro. Nevertheless, optic disc and stalk tissues are not reported in any of these structures.

We hypothesize that coordinated specification from the anterior ectodermal epithelium via morphogen gradients leads to self-organization of telencephalic and ocular tissues, including optic disc- and stalk-like tissues that provide guidance cues for RGC axon growth and pathfinding. In support of this hypothesis, we generated self-formed human telencephalon-eye organoids that comprise concentric zones of anterior ectodermal progenitors (CONCEPT), including tissues mimicking FOXG1+ telencephalon, PAX2+ optic stalk, PAX2+ optic disc, VSX2+ neuroretina, and PAX6+ multi-lineage tissues along the center-periphery axis. We refer these tissues as telencephalon, optic stalk, optic disc, and neuroretina for the sake of gene marker expression, tissue functions, and the ease of description; we do not claim these tissues represent in vivo tissues in all aspects. We call this structure as an organoid since it displays a well-organized structure with cell identities mimicking those tissues in vivo. In CONCEPT organoids, early differentiated RGCs grew their axons toward and then along a path defined by adjacent PAX2+ optic-disc cells. Single-cell RNA sequencing of CONCEPT organoids not only confirmed telencephalic and ocular identities but also discovered expression signatures of cell clusters. We found that PAX2+ optic-disc cells differentially expressed FGF8 and FGF9; inhibition of FGF signaling with an FGFR inhibitor during early RGC differentiation drastically decreased RGCs somas and nearly ablated directional axon growth. Using cell-surface marker CNTN2 identified in our single-cell RNA-sequencing, we developed a one-step method for isolating human electrophysiologically-excitable RGCs under a native condition. Our studies provide deeper insight into the coordinated specification of telencephalic and ocular tissues to ensure guided RGC axon growth in humans and establish resources for studying neurodegenerative diseases such as glaucoma.

## Results

### Generation of a telencephalon-eye organoid that is composed of concentric zones of anterior ectodermal progenitors (CONCEPT)

The telencephalon and eye in mammals are originated from adjacent fields at the anterior neuroepithelium ^1^. Morphogenesis of these embryonic fields leads to the formation of telencephalon, optic stalk, optic disc, and neuroretina along an axis. Early differentiated retinal ganglion cells (RGCs) in the neuroretina grow axons toward the optic disc and then along the optic stalk to reach the brain. How these telencephalic and ocular tissues are specified coordinately to ensure directional RGC axon growth is unclear in humans.

Cysts are hollow spheres of an columnar epithelium induced from human pluripotent stem cells through embedding hESC sheets in Matrigel; they are used to generate retinal cells ^28,33,47^. Nevertheless, developmental potentials of cysts have not been characterized.

**Figure 1.**
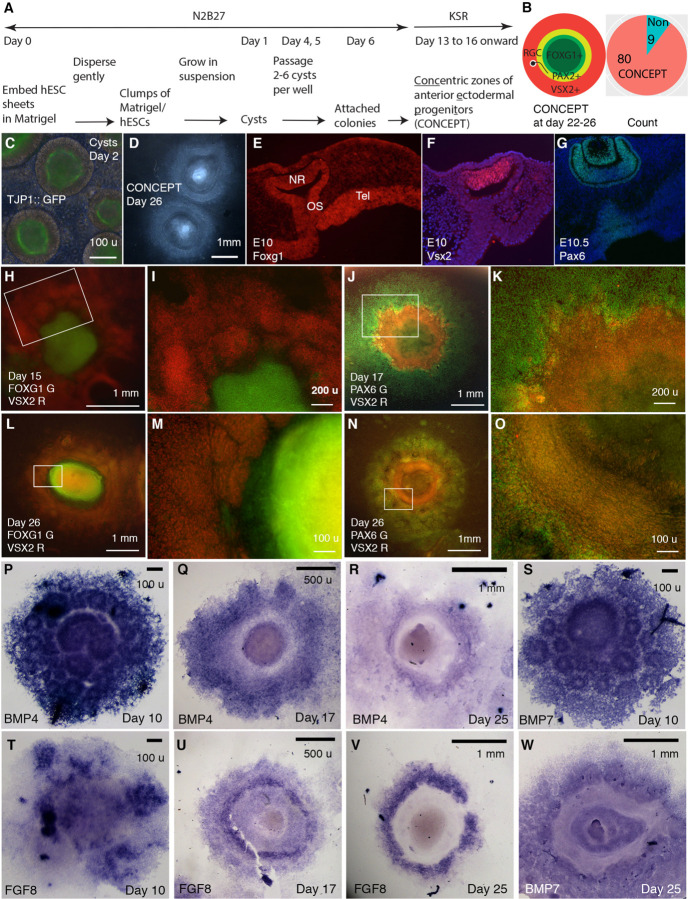
Generation of a telencephalon-eye organoid composed of concentric zones of anterior ectodermal progenitors (CONCEPT). **(A)** A scheme of the procedure. **(B)** A diagram of CONCEPT organoids showing concentric zones of the anterior ectodermal progenitors. See also Figs.1, 2, 3, 5, S1, S10. **(C)** Morphology of cysts at day 2 showing the epithelial structure indicated by the apical reporter TJ::GFP at the lumen. **(D)** Morphology of CONCEPT organoids at day 26. **(E-G)** Expression of telencephalon (Tel) marker Foxg1, neuroretinal (NR) markers Vsx2 and Pax6 in mouse eyes at E10–10.5. Rostral optic stalk (OS) connected the telencephalic vesicle to the optic cup. **(H-O)** FOXG1+ telencephalic progenitors, VSX2+ and/or PAX6+ retinal progenitors formed concentric zones in CONCEPT organoids. N > 5 experiments. **(P-W)** In CONCEPT organoids, morphogens FGF8, BMP4, and BMP7 mRNA expression started at early stages and subsequently formed circular gradients. N > 5 experiments. Scale bars, 100 µm (C, E, M, O, P, S, T), 200 µm (I, K), 500 µm (Q, U), 1 mm (D, H, J, L, N, R, V, W).

Using the cysts, here we generate a telencephalon-eye organoid that is composed of concentric zones of anterior ectodermal progenitors (CONCEPT) from cysts (Fig. 1A, B). Cysts were generated as previously described ^28,33^. The epithelial structure of cysts was demonstrated by apically localized TJP1::GFP reporter at the lumen (Fig. 1C), consistent with previous findings ^28,47^. To assess developmental potentials of cysts, individual cysts were manually picked and then seeded onto the Matrigel-coated surface at low densities (Fig. 1A). After attaching to the culture surface, cysts initially grew as dome-shaped individual colonies. Subsequent culture of the colonies in a KSR medium (see Materials and Methods) led to the self-formation of concentric zones of anterior ectodermal progenitors: an elevated central zone surrounded by multiple zones; this morphology was distinguishable under a steoromicroscope (Fig. 1D) or an inverted microscope. CONCEPT structures were highly reproducible, which is demonstrated by consistent gene expression profiles of multiple CONCEPT organoids in whole culture wells (Fig. S1). If multiple cysts were fused together or cysts for seeding were too big or small, CONCEPT structures were affected or incomplete. CONCEPT structures expressed gene markers for telencephalon and eye in concentric patterns. In mice, Foxg1 is specifically expressed in the E10 telencephalon at high levels; it was also expressed in the optic stalk, invaginating optic cup, and lens at lower levels, with the rostral optic stalk connecting the telencephalic vesicle to the invaginating optic cup (Fig. 1E) ^6^; Vsx2 is specifically expressed in the E10.5 neuroretina (Fig. 1F); Pax6 is specifically expressed in the E10.5 neuroretina, RPE, lens vesicles, and surface ectoderm (Fig. 1G) ^13^. In CONCEPT structures, the expression of FOXG1, VSX2, and PAX6 generally exhibited concentric patterns spanning from the center to the periphery (Fig. 1H-O). Similar to FOXG1, telencephalon marker EMX2 ^48^ was also expressed at the central region (Fig. S1F). PAX6 was expressed in multiple concentric zones at distinct levels, mimicking its expression patterns in multiple ocular tissues in E10.5 mouse eyes (Fig. 1G, J, K, N, O). Therefore, the CONCEPT structure is a telencephalon-eye organoid mimicking telencephalic and ocular tissues in the aspects of cell types and patterning at early stages.

Morphogens FGFs and BMPs play crucial roles in patterning the forebrain and eye in vivo. In mice, Fgf8 is specifically expressed at the rostral forebrain at early stages, induces Foxg1 expression ^19^, and regulates telencephalic patterning in a dose-dependent manner ^20^. Bmp4 and Bmp7 are expressed in the dorsomedial telencephalon, optic vesicles, and presumptive lens placodes ^49–52^. Bmp4 is required for lens induction ^50^, and Bmp7 is required for proper patterning of the optic fissure ^53^. In CONCEPT telencephalon-eye organoids, FGF8, BMP4, and BMP7 were expressed in attached cell colonies starting from early stages (Fig. 1P, S, T) and subsequently exhibited circular patterns (Fig. 1R, V, W). At day 10, the expression of BMP4 and BMP7 delineated multiple rings, with a big ring mostly at the center surrounded by numerous small rings (Fig. 1P, S). These observations suggest that the attachment of a single-lumen cyst to the culture surface caused differences in cell behaviors: some cells separated from the original cyst and migrated peripherally; some of the separated cells formed small ring-like structures; the cells that remained at the center formed a big ring-like structure. At day 17, circular patterns of BMP4 and FGF8 emerged (Fig. 1Q, U). At day 25, BMP4, FGF8, and BMP7 clearly exhibited circular patterns (Fig. 1R, V, W). Hence, FGFs and BMPs spontaneously form circular gradients, which would dictate coordinated cell specification in CONCEPT telencephalon-eye organoids.

### Early differentiated RGCs grow directional long axons toward and then along a path defined by PAX2+ cell populations in CONCEPT telencephalon-eye organoids

To assess cell differentiation in CONCEPT telencephalon-eye organoids, we examined marker expression for RGCs, the first type of cells that differentiate in the neuroretina. In mice, transcription factor Pou4f2 is expressed in the early differentiated RGCs and required for the development of a large set of RGCs ^54^. Tubb3 is expressed in the somas and axons of differentiating RGCs.

**Fig. 2.**
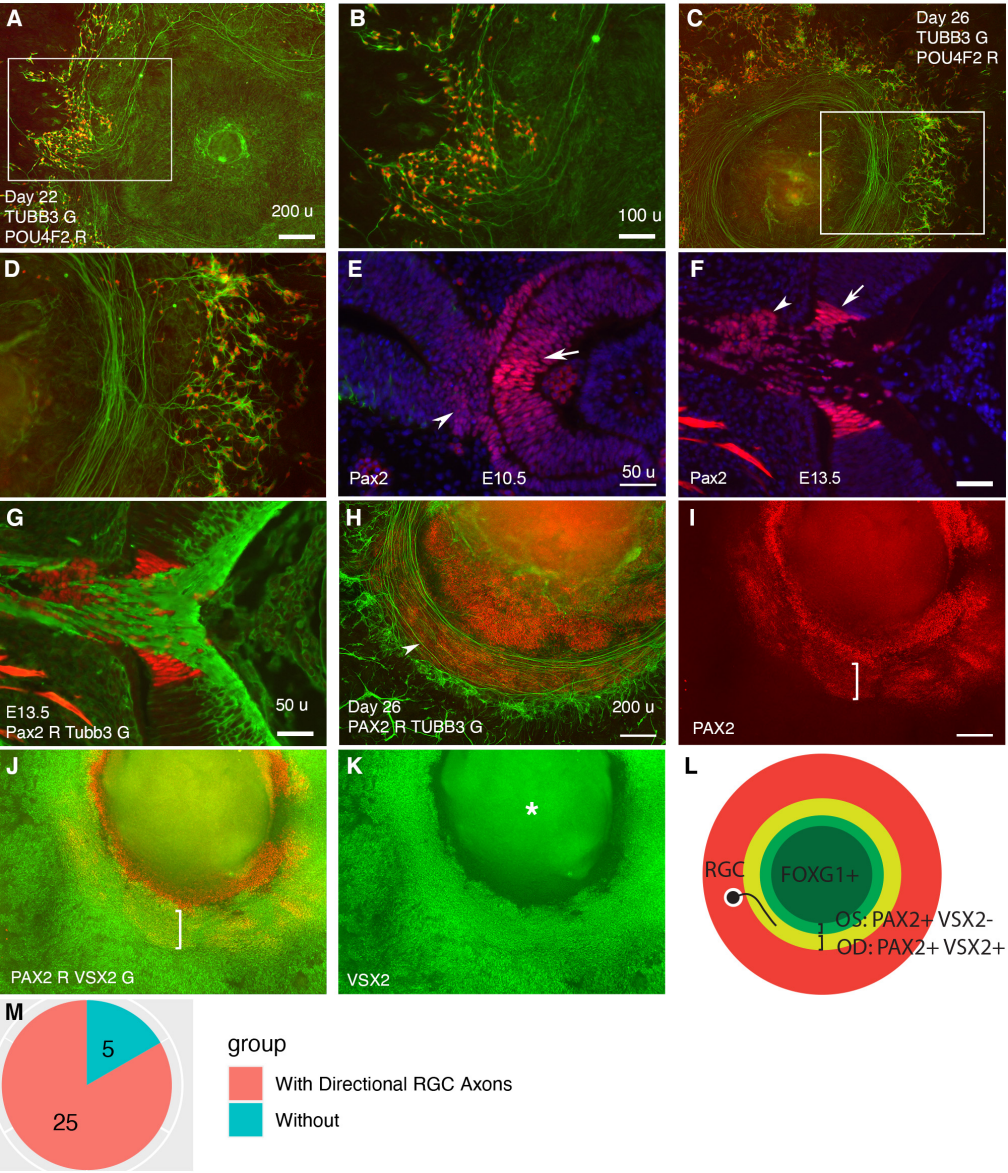
Retinal ganglion cells (RGCs) grow axons toward and then along a path defined by PAX2+ cell populations in CONCEPT telencephalon-eye organoids. N > 5 experiments. **(A-D)** POU4F2+ RGCs grew TUBB3+ axons toward and then along a path with a circular or a portion of circular shape. **(E, F)** In mice, Pax2 was expressed in central regions of the retina and optic stalk at E10.5 (E) and in the optic disc and optic stalk at E13.5 (F). Tubb3+ axons from the initial RGCs grew toward the optic disc, exited the eye, and navigated within the optic stalk (G). **(H-L)** In CONCEPT organoids at day 26, TUBB3+ RGC axons grew toward and then along a path defined by an adjacent PAX2+ VSX2+ cell population (arrowhead in H, brackets in I, J); the PAX2+ VSX2− cell population sets up an inner boundary of RGC axon growth. **(L)** A diagram summarizing RGC axon growth, PAX2+ optic disc (OD), and PAX2+ optic stalk (OS) in CONCEPT organoids. The area labeled by the asterisk may appear as false signals in a low-resolution printout but it is clearly a background in digital display. **(M)** A count of CONCEPT organoids showing directional retinal ganglion cell axons. Scale bars, 50 µm (E, F, G), 100 µm (B), 200 µm (A, H, I).

In CONCEPT organoids, POU4F2 and TUBB3 were detectable as early as day 17 and increased to higher levels at day 22. RGC somas were marked by POU4F2 and TUBB3 co-expression; RGC axons were marked by TUBB3 expression. Interestingly, RGCs grew directional long axons that followed a circular path (Fig. 2A, B). The circular path of RGC axon outgrowth became more evident in organoids at day 26 (Fig. 2C, D). In mice, the initially differentiated RGCs are adjacent to the optic disc; their axons grow towards the optic disc to exit the eye and then navigate within the optic stalk to reach their targets in the brain; the optic disc and stalk specifically express Pax2 at distinct levels (Fig. 2E-G) ^17,55,56^; presumptive optic disc cells expressed VSX2 whereas optic stalk cells do not ^13^ (Fig. 1F; 2E). In CONCEPT organoids, there were two PAX2+ cell populations that formed two adjacent rings (Fig. 2H-L). POU4F2+ RGCs grew TUBB3+ axons toward and then navigated along the adjacent PAX2+ VSX2+ cell population (Fig. 2H-L), mimicking axon growth from the initial RGCs toward the PAX2+ optic disc in vivo. Meanwhile, PAX2+ VSX2− cell population at the inner zone set up an inner boundary of the path for RGC axon growth, mimicking PAX2+ optic stalk that spatially confines RGC axon growth in vivo. Based on these findings, we designate PAX2+ VSX2+ cells and PAX2+ VSX2− cells as the optic disc and optic stalk, respectively. Taken together, our findings demonstrate that RGCs grow directional axons toward and then along a path defined by PAX2+ cell populations in CONCEPT telencephalon-eye organoids.

### CONCEPT telencephalon-eye organoids contain lens cells that undergo terminal differentiation

**Fig. 3.**
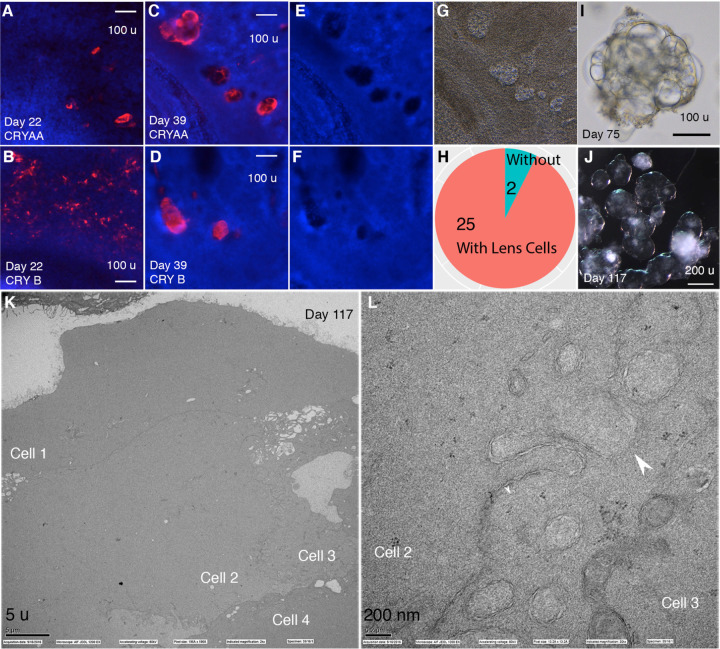
CONCEPT telencephalon-eye organoids contain lens cells that undergo terminal differentiation. N > 5 experiments. **(A-H)** In CONCEPT organoids, lens markers CRYAA and CRY B were expressed at day 22 (A, B) and day 39 (C, D). Lens cells were not stained by DAPI (E, F); they exhibited a crystal-like shape (G). A count of CONCEPT organoids with these lens cells is shown (H). **(I, J)** When CONCEPT organoids were detached using Dispase at around day 28 and grown in suspension, crystal-like clusters were found (I) and survived for months (J). **(K-L)** Crystal-like lens clusters were free of organelles and exhibited ball-and-socket structures (K, L), as revealed by electron microscopy. Scale bars, 100 µm (A, B, C, D, I), 200 µm (J), 5 µm (K), 200 nm (L).

CONCEPT telencephalon-eye organoids at stages around day 25 contained transparent structures reminiscent of the ocular lens. To determine their cell identity, we performed immunostaining. Starting at day 22, lens markers CRYAA and CRY B were found (Fig. 3A, B) and continuously expressed in the transparent cell structures (Fig. 3C, D). Interestingly, these transparent structures were not stained by DAPI (Fig. 3C-F), indicating denucleation in these lens cells. When CONCEPT organoids were detached using Dispase at around day 28 and continuously grown as suspension cultures, crystal-like clusters with fused transparent spheres were found, and they continuously survived for months (Fig. 3I, J). In mice, terminally-differentiated lens fiber cells are free of organelles and featured by specialized interlocking cell membrane domains shown as ball-and-sockets and protrusions ^57^. Using transmission electron microscopy, we found that the crystal-like lens clusters were free of organelles and exhibited ball-and-socket structures (Fig. 3K, L). Taken together, our findings demonstrate that CONCEPT telencephalon-eye organoids contain lens cells that undergo terminal differentiation; FGFs in CONCEPT organoids likely promote terminal lens differentiation as seen in other settings ^58^.

### Single-cell RNA sequencing analysis identifies telencephalic and ocular cell populations in CONCEPT telencephalon-eye organoids

**Fig. 4.**
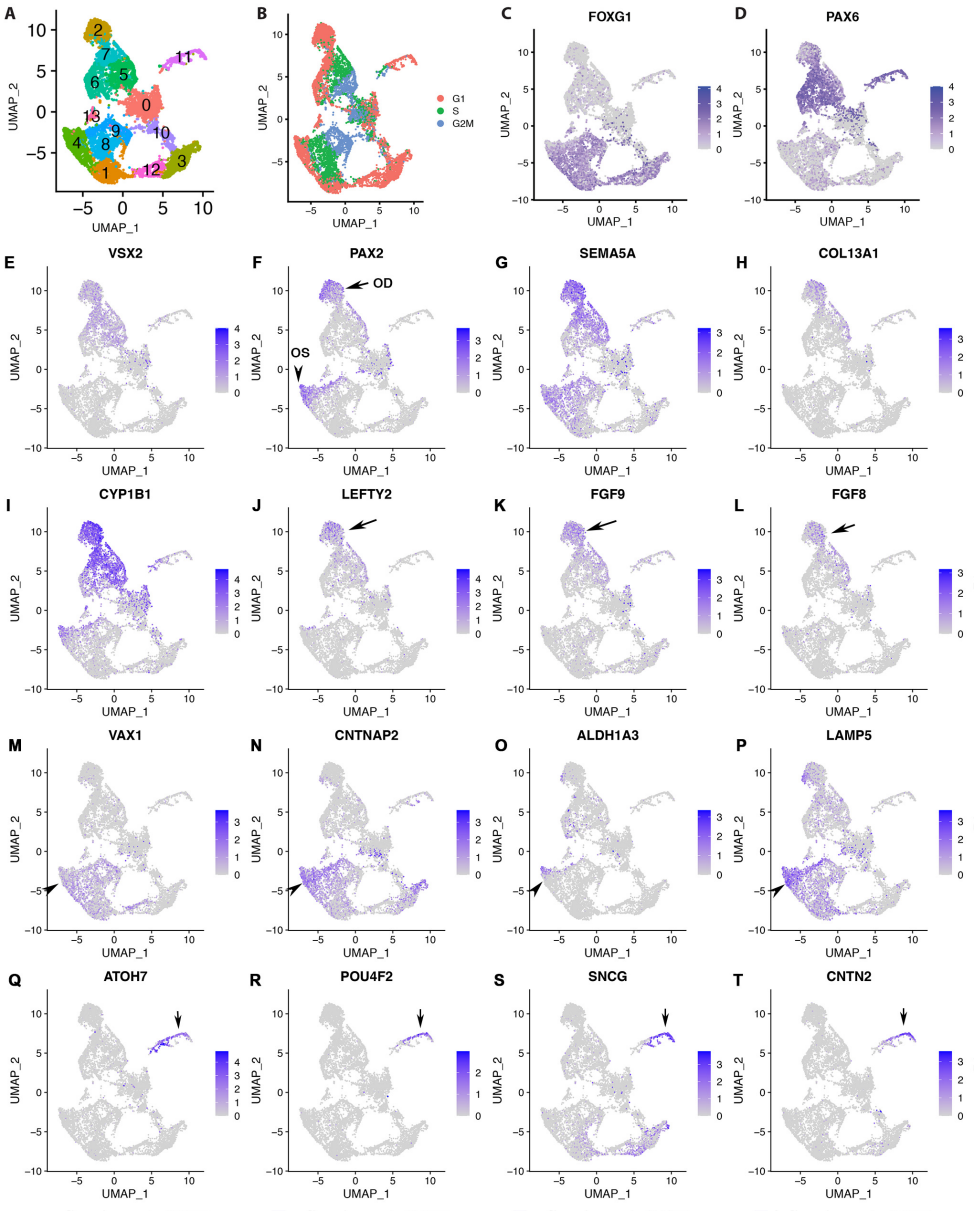
Cell clustering analysis identifies telencephalic cells, ocular cells, and two PAX2+ cell populations that mimic the optic disc and optic stalk, respectively. See also suppl. Figs. S2-S8. CONCEPT organoids at day 24 were used for single-cell RNA sequencing and analyzed using Seurat (v3.2.0). **(A)** Identification of 14 cell clusters. **(B)** Cell cycle phases as shown as cell cycle scores. **(C)** FOXG1 expression marked telencephalic cells. **(D, E)** The expression of PAX6 and/or VSX2 marked retinal cells. **(F)** PAX2+ cells were found in two major cell populations: PAX2+ VSX2+ cells were assigned as the optic disc (OD), whereas PAX2+ FOXG1+ VSX2− cells were assigned as the optic stalk (OS). **(G)** The expression of the optic disc/stalk marker SEMA5A. **(H-L)** The expression of major DEGs in cluster 2, the major cell population that mimic the optic disc. **(M-P)** The expression of major gene markers for PAX2+ VSX2− optic stalk cells. **(Q-T)** Identification of glycosylphosphatidylinositol (GPI)-anchored cell membrane protein CNTN2 as a specific marker for developing human RGCs. Cluster 11 differentially expressed RGC markers, including CNTN2.

To fully characterize cell populations in CONCEPT organoids and the mechanisms underlying RGC axon pathfinding, we performed 10x single-cell RNA sequencing of the organoids at day 24, around the stage when RGCs grew long axons toward and along the PAX2+ VSX2+ cell population. Cell Ranger mapping showed that 11158 single cells were sequenced at a depth of 27,842 reads and 2,967 genes per cell, and the dataset were then analyzed using Seurat (v3.2.0) ^59^. Cell filtration (nFeature_RNA > 200 & nFeature_RNA < 6000 & percent.mt < 20) resulted in 10218 cells with high quality. Cell clustering grouped cells into 14 clusters (Fig. 4A; Tab. 1). In addition, cell cycle scores were plotted using an established method (Fig. 4B) ^60^. Several cell clusters, *e.g.,* clusters 4, 8, 9, were separated along cell cycle scores (Fig. 4A, B), indicating the differences in cell cycle phases across these cell clusters.

We next assessed cell identities using known markers. Mesoderm, endoderm, and neural crest are identified by a group of gene markers ^61–64^. In CONCEPT organoids, gene markers for mesoderm (TBXT, GATA2, HAND1), endoderm (GATA1, GATA4, SOX17), and neural crest (SNAI1, SOX10, FOXD3) were not expressed (Fig. S2). In contrast, gene markers for the anterior neuroectoderm were widely expressed. Telencephalic progenitor marker FOXG1 was expressed in clusters 10, 3, 12, 1, 4, 8, 9, 13; retinal progenitor markers PAX6 and VSX2 were expressed in clusters 2, 7, 5, 6, 11 (Fig. 4C-E). Cluster 0 was defined by a group of negative markers (Fig. S3); they expressed both FOXG1+ telencephalic cells and PAX6+ and/or VSX2+ retinal cells. In contrast to telencephalon and retinal markers, diencephalon markers (GBX2, WNT3, SOX14) ^65,66^ and midbrain/hindbrain markers (EN2, PAX7, TFAP2B) ^61^ were rarely expressed (Fig. S4). Lens markers CRYAA and FOXE3 were expressed in a very small cell population; those denucleated lens cells were not captured in the scRNA-seq since the absence of nucleus prevented active mRNA transcription. Therefore, lens cells did not form a separate cluster probably due to their very low abundance. Taken together, these findings indicate that CONCEPT telencephalon-eye organoids at day 24 are mostly composed of FOXG1+ telencephalic cells and PAX6+ and/or VSX2+ retinal cells.

We next characterized the FOXG1+ telencephalic cells via assessing differentially expressed genes (DEGs). DEGs in clusters 4, 8, 9, 1 included POU3F3, RGMA, EDNRB, and SOX3, which orthologs in mice are expressed in the pallium. DEGs in clusters 12, 3, 10 included DLX2, RGS16, DLX1, DLL1, DLX6-AS1, NEFL, DCX, and RTN1, which orthologs in mice are expressed in the subpallium (Figs. S5, S6). Therefore, FOXG1+ telencephalic cells in CONCEPT organoids at day 24 comprise cell clusters in both the pallium and subpallium.

PAX6+ and/or VSX2+ retinal cells were also grouped into several clusters. VSX2 was expressed in clusters 2, 7, 5, and 0, with cluster 2 at G1 phase, cluster 7 in G1 and S phases, and cluster 5 in S and G2M phases (Fig. 4A, B, E). Cluster 6 did not VSX2 but expressed PAX6 and was at the G1 phase, suggesting cells in cluster 6 exited the retinal progenitor cell state and underwent cell differentiation (Fig. 4A, B, D, E). Consistently, cluster 6 differentially expressed RPE markers, e.g., PMEL, HSD17B2, DCT, and MITF (Fig. S7), indicating they were mostly differentiating RPE. Cluster 11 did not express VSX2; instead, it expressed PAX6 and was at the G1 phase (Fig. 4A, B, D, E), suggesting that cells in cluster 11 exited the retinal progenitor cell state and underwent cell differentiation.

Taken together, our single-cell RNA sequencing analysis of CONCEPT telencephalon-eye organoids at day 24 confirms their telencephalic and ocular identities, establishing a valuable transcriptomic dataset for mechanistic studies.

### Identification of two PAX2+ cell populations that mimic to the optic disc and optic stalk, respectively, in the scRNA-seq dataset of CONCEPT telencephalon-eye organoids

**Fig. 5.**
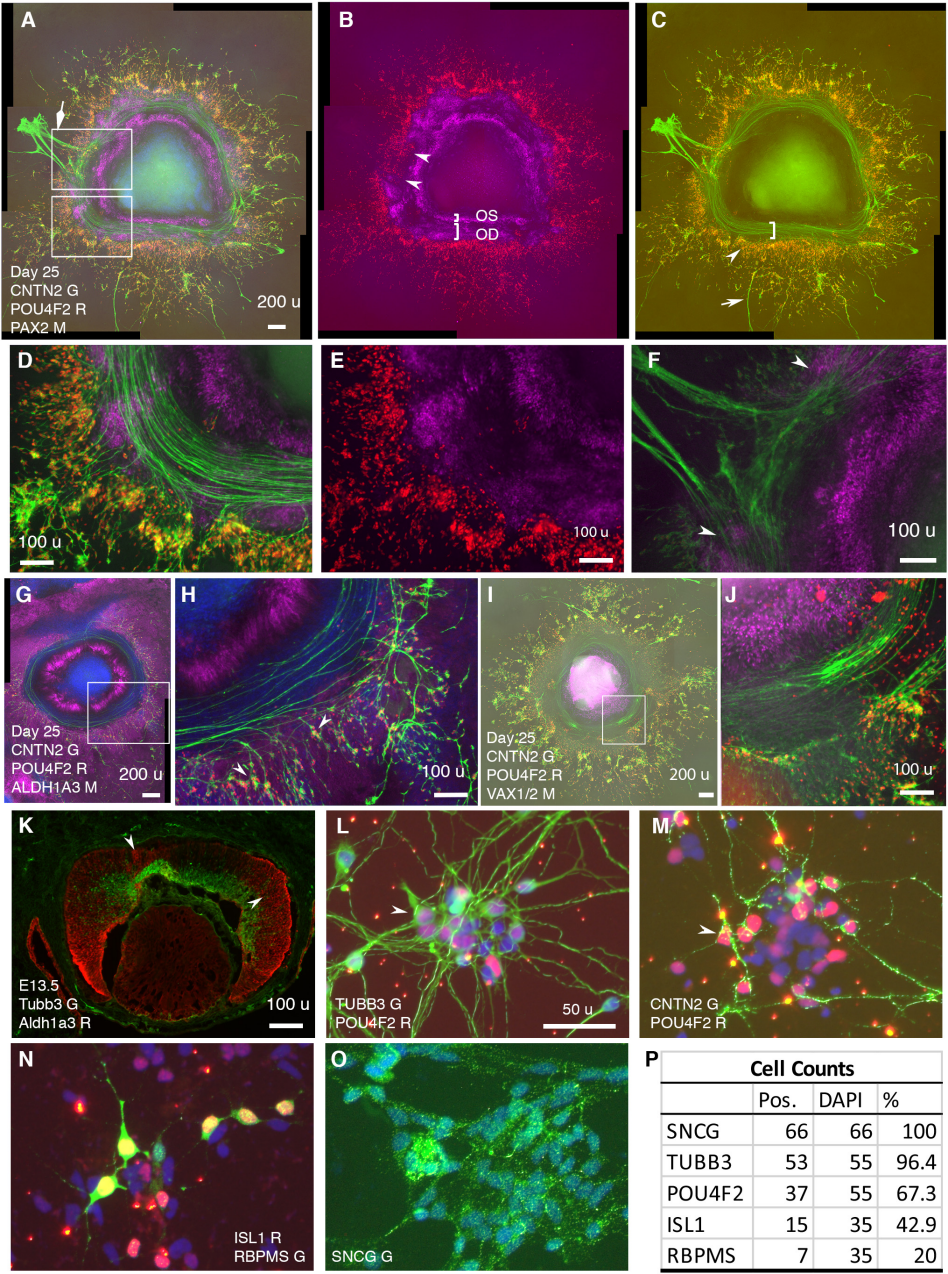
RGCs grow CNTN2+ axons toward and then along a defined path in CONCEPT telencephalon-eye organoids and can be isolated in one step via CNTN2 in a native condition. N > 5 experiments. **(A-F)** PAX2+ cells formed two concentric zones mimicking the optic stalk (OS) and optic disc (OD), respectively (A-C; high magnifications in D, E). POU4F2+ RGCs grew CNTN2+ axons toward and then along a path defined by adjacent PAX2+ optic-disc cells (A-E). RGCs at a few hundreds of micrometers away from PAX2+ optic-disc cells grew axons centrifugally (arrow in C). At regions where there was a gap in PAX2+ optic-disc cells, CNTN2+ RGC axons exited the circular path and grew centrifugally (diamond arrowhead in A, double arrowheads in B and F). PAX2+ optic-stalk cells set up an inner boundary for RGC axon growth. **(G, H)** PAX2+ optic-disc cells did not express ALDH1A3; the cells that set up the boundaries of the path highly expressed ALDH1A3. **(I, J)** Cells that set up the inner boundary for RGC axon growth expressed VAX1/VAX2. **(K)** In E13.5 mouse eye, Aldh1a3 was highly expressed in the peripheral retina and a small region in the optic disc. Aldh1a3 expression was at low or nearly absent in the central retina. **(L-P)** One-step isolation of RGCs. RGCs from floating retinal organoids at day 41 (L, M) and day 70 (N-O) were dissociated into single cells using Accutase and then isolated using MACS via CNTN2 for 10-day growth. Isolated RGCs expressed POU4F2 and grew TUBB3+ neurites in random directions (L). RGCs also expressed CNTN2 (M), ISL1 (N), RBPMS (N), and SNCG (O); positive cells were counted (P). Scale bars, 200 µm (A,G,I), 100 µm (D-F,H,J,K), 50 µm (L).

To identify PAX2+ cell populations that defined the path for RGC axon outgrowth, we examined PAX2 expression in the dataset. PAX2 was mostly expressed in cluster 2 and subsets of clusters 7, 5, 0, 4, 8, and 9 (Fig. 4A, F). Since clusters 2, 7, 5, and 0 expressed VSX2 (Fig. 4E, F), we deduced that PAX2+ VSX2+ cells in cluster 2 and subsets of clusters 7, 5, and 0 corresponded to those PAX2+ VSX2+ cells that defined the path for RGC axon growth (Figs. 2H-L). Therefore, these PAX2+ VSX2+ cells in the dataset of single-cell RNA sequencing were assigned as the optic disc (OD) (Fig. 4F). PAX2+ VSX2+ cells also expressed optic disc and optic stalk marker SEMA5A (Fig. 4F, G) ^67^. We particularly focused on cluster 2 since PAX2+ VSX2+ cells were mostly found in this cluster. Interestingly, COL13A1 was differentially expressed in cluster 2 (Fig. 4H; Fig. S10); in situ hybridization indicates that COL13A1 is specifically expressed in the optic disc of human fetal retinas ^68^. These findings firmly support that PAX2+ VSX2+ cells in CONCEPT telencephalon-eye organoids corresponded to optic disc cells in human fetal retinas. Cluster 2 also differentially expressed glaucoma gene CYP1B1, signaling molecules LEFTY2, FGF9, and FGF8 (Fig. 4I-L; Fig. S10). The expression of PAX2, SEMA5A, CYP1B1, LEFTY2, FGF9, and FGF8 in CONCEPT organoids was validated using in situ hybridization (Figs. 1T-V; 5B-E; S10).

In contrast, PAX2+ VSX2− cells in subsets of clusters 4, 8, 9 (Fig. 4A, E, F) expressed the optic stalk marker VAX1 (Fig. 4F, M); these cells corresponded to those PAX2+ VSX2− cells that set up the inner boundary of the path for RGC axon growth in CONCEPT organoids (Fig. 2H-L). Therefore, PAX2+ VSX2− cells in subsets of clusters 4, 8, 9 were assigned as the optic stalk (OS) (Fig. 4F). PAX2+ VSX2− optic stalk cells also differentially expressed CNTNAP2, ALDHA3, and LAMP5 (Fig. 4N-P), which orthologs in mice are expressed in the optic stalk/nerve (Fig. S8). Compared to PAX2+ VSX2+ optic disc cells, PAX2+ VSX2− optic stalk cells were closer to telencephalic cells in both the UMAP plot (Fig. 4M-P; Fig. S5A-D) and their positioning in CONCEPT organoids (Fig. 2H-L), mimicking relative positions of telencephalon, optic stalk, and optic disc along the midline-periphery axis in E10.5-E13.5 mouse embryos (Figs. 1E, 2E-G). Consistently, several markers for PAX2+ VSX2− optic stalk cells were also expressed in the telencephalon (Fig. S8). Collectively, we identify two PAX2+ cell populations that mimic the optic disc and optic stalk, respectively, in the scRNA-seq dataset of CONCEPT telencephalon-eye organoids.

### Identification of glycosylphosphatidylinositol (GPI)-anchored cell membrane protein CNTN2 as a specific marker for developing human RGCs

To identify RGCs in the dataset, we checked the expression of RGC markers. We found that RGC markers ATOH7, POU4F2, SNCG, and CNTN2 were differentially expressed in cluster 11, indicating its RGC identity (Fig. 4A, 4Q-T). CNTN2 caught our attention because it is a GPI-anchored cell membrane protein, which may be used as a native marker for RGC isolation. In literature, Cntn2 is specifically expressed in developing RGCs in mice and chicks ^69,70^.

In CONCEPT at day 25, CNTN2 exhibited an expression pattern very similar to that of TUBB3 (Figs. 2, 5). CNTN2 was found in the cell membrane of RGCs that expressed POU4F2 in the cell nucleus (Fig. 5A, D). PAX2+ cells formed inner and outer concentric zones, mimicking the optic stalk and optic disc, respectively. POU4F2+ RGCs formed a dense circular zone adjacent to PAX2+ optic disc cells; POU4F2+ RGCs were sparse in more peripheral areas (Fig. 5B). These findings indicate that early differentiated RGCs were adjacent to PAX2+ optic disc cells. Interestingly, RGCs in the dense POU4F2+ zone grew CNTN2+ axons toward and then along the path defined by adjacent PAX2+ optic disc cells (Fig. 5A-E). In areas where there were a gap in PAX2+ optic disc cells, CNTN2+ axons exited the circular path (diamond arrow in Fig. 5A; a gap between two arrowheads in Fig. 5B, F). In regions where POU4F2+ RGCs were a few hundreds of micrometers away from PAX2+ optic disc cells, RGCs grew axons in centrifugal directions (arrow in Fig. 5C). Very similar findings were found using hiPSCs (Fig. S9), indicating the reproducibility of CONCEPT organoids in multiple cell lines. PAX2+ optic disc cells did not express ALDH1A3; instead, the path for RGC axon growth was bordered by two cell populations that highly expressed ALDH1A3 (Fig. 5G-H). In mice, Aldh1a3 expression was low in differentiating RGCs in the central retina but high in peripheral retinal progenitors and optic stalk (Fig. 5K) ^71^, consistent with ALDH1A3 expression in CONCEPT organoids (Fig. 5G, H). In mice, Vax1 and Vax2 are expressed in the optic stalk and ventral retina and are jointly required for the optic stalk development ^55,72^. In CONCEPT organoids, cells that set up the inner boundary for RGC axon growth also expressed optic stalk marker VAX1/2 (Fig. 5I, J; the antibody recognizes both VAX1 and VAX2). Taken together, our data demonstrate that CNTN2 is a specific cell-surface marker for differentiating human RGCs; RGCs grow axons toward and then along a path that is defined by adjacent PAX2+ VSX2+ ALDH1A3− optic disc cells.

### One-step isolation of developing human RGCs via native marker CNTN2

Since cell surface protein CNTN2 was specifically expressed in developing human RGCs, we sought to test whether CNTN2 can be used as a biomarker for isolating human RGCs under a native condition. To that goal, retinal organoids in suspension culture on days 41 – 70 were dissociated using Accutase to generate a single cell suspension, which was then subject to magnetic-activated cell sorting (MACS) with an antibody against CNTN2. From 100 retinal organoids on day 41 to 48, around 385,000 RGCs were isolated. Isolated RGCs were plated onto Matrigel-coated chamber slides for 10-day adherent culture. These cells exhibited neuronal morphology and widely expressed RGC markers TUBB3 and POU4F2 (Fig. 5L). Isolated RGCs tended to form clusters in culture. In contrast to directional axon growth found in CONCEPT organoids, isolated RGCs grew neurites in random directions (Fig. 5L), indicating the differences in axon pathfinding cues between CONCEPT organoids and isolated RGC cultures. RGC neurites were also marked by CNTN2 expression (Fig. 5M). A portion of isolated RGCs expressed POU4F2 but not CNTN2 (Fig. 5M), indicating that CNTN2 was downregulated in dissociated RGC cultures. Isolated RGCs also expressed RGC markers ISL1, RBPMS, and SNCG (Fig. 5N-P). Collectively, we have developed a one-step method for the isolation of differentiating human RGCs in a native condition.

### Isolated RGCs exhibit electrophysiological signature of excitable cells

**Fig. 6.**
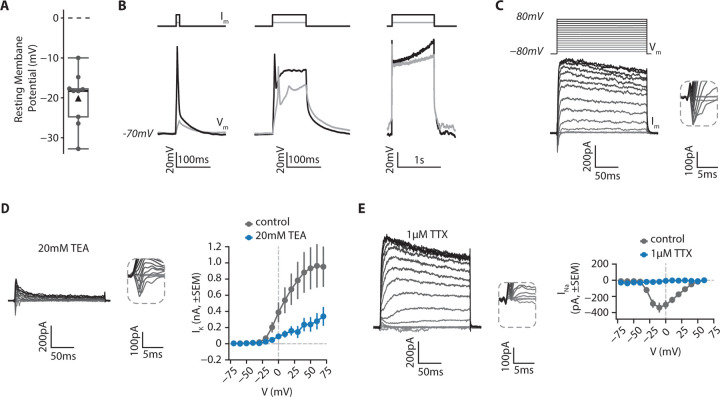
Electrophysiological features of RGCs. RGCs from retinal organoids on day 48 were isolated using MACS via a CNTN2 antibody and then grown on polymer coverslips in a chamber slide for 20–25 days before whole-cell patch clamp recordings. **(A)** Resting membrane potential of RGCs. *Black triangle: average; black line: median; box: interquartile range, n=9*. **(B)** RGCs can fire action potentials. Cells were patched in current-clamp mode. A steady current (*I*_*m*_) was injected to maintain the membrane potential at −70mV and depolarizing current steps of 10ms (*left*), 100ms (*middle*) or 1s (*right*) were injected to elicit action potentials (*V*_*m*_). **(C)** RGCs show functional voltage-gated currents. Cells were recorded in voltage-clamp (holding=−80mV) and depolarizing voltage steps (200ms, +10mV steps up to 80mV, *V*_*m*_) were applied to record inward and outward voltage-gated currents (*I*_*m*_). *Inset*: zoom on inward currents. **(D)** Outward currents are primarily due to voltage-gated potassium channels. *Left*, representative example of a current-voltage experiment performed in presence of 20mM Tetraethylammonium (TEA), a blocker of voltage-gated potassium channels. *Inset*: zoom on inward currents. *Right*, amplitude of potassium current as a function of membrane potential (*mean±SEM; n*_*control*_*=5, n*_*TEA*_*=5*). **(E)** Inward currents result from activity of voltage-gated sodium channels. *Left*, representative example of a current-voltage experiment performed in presence of 1µM Tetrodotoxin (TTX), a blocker of voltage-gated sodium channels. *Inset*: zoom on inward currents. *Right*, amplitude of sodium current as a function of membrane potential (*mean±SEM; n*_*control*_*=5, n*_*TTX*_*=3*).

In order to determine the functional properties of isolated RGCs in culture, we examined their electrophysiological properties using whole-cell patch clamp recordings (Fig. 6, *see* Methods). Using current-clamp configuration, we found that RGCs displayed had a hyperpolarized resting membrane potential (*mean ±SD: −20.1 ±6.4mV*, Fig. 6A). When cells were held at −70 mV by current injection, in most cases (6/9), depolarizing currents steps could trigger an action potential, which were often followed by a depolarization plateau if the current was injected for more than 10ms (Fig. 6B). In voltage clamp recordings we examined the nature of the voltage-gated conductances (*see* Methods). From a holding membrane potential of −80 mV, both inward and outward currents (*I*_*m*_) were observed in response to depolarizing voltage steps (*V*_*m*_, Fig. 6C). Outward currents were primarily mediated by voltage-gated potassium channels, as application of 20mM TEA significantly reduced their amplitude (*mean I*_*K*_ *±SD at 60mV; 964±537 pA in control; 279±201 pA in TEA, U=24.0; p=0.019, two-sided MannWhitneyU test*, Fig. 6D). Conversely, 1µM TTX abolished all inward currents, demonstrating that they were mediated by activation of voltage-gated sodium channels (*mean I*_*Na*_ *±SD at −10mV; −338.2±121 in control; −18.3±13 pA in TTX; U=0.0; p=0.035, two-sided MannWhitneyU test*, Fig. 6E). Taken together, these results indicate that isolated RGCs exhibit functional features traditionally found in excitable cells, such as in neurons.

### FGF signaling mediated by FGFRs is required for early RGC differentiation and directional axon growth in humans

**Fig. 7.**
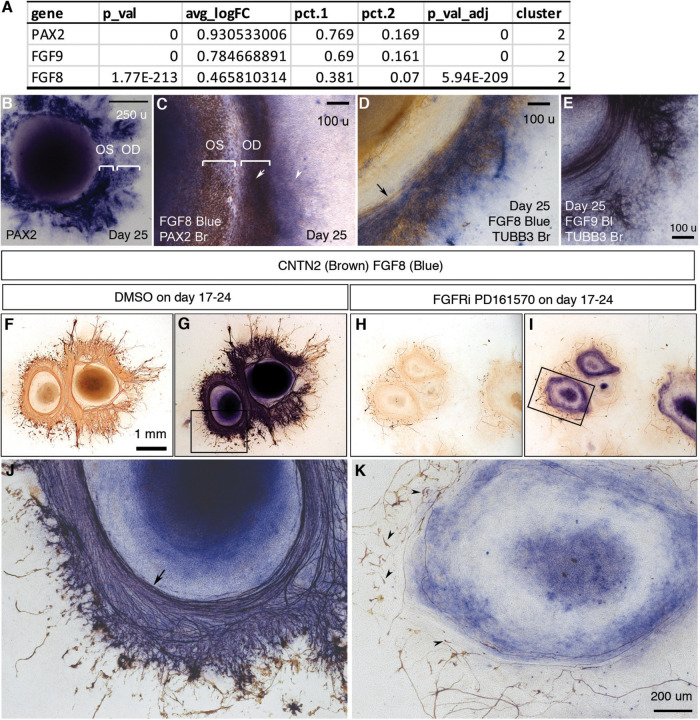
FGF signaling mediated by FGFRs is required for early RGC differentiation and directional axon growth. **(A)** PAX2, FGF9, and FGF8 are differentially expressed in cluster 2, the major component of PAX2+ optic-disc cells. **(B)** PAX2 mRNA expression in CONCEPT organoids on day 25. Two PAX2+ concentric zones corresponding to the optic stalk (OS) and optic disc (OD) are labeled. **(C)** Dual-color immunocytochemistry indicates the co-localization of FGF8 and PAX2 in the optic-disc zone of CONCEPT organoids on day 25. **(D-E)** TUBB3+ axons grew towards and then along the cells that expressed high levels of FGF8 (D) and FGF9 mRNA (E) in CONCEPT organoids on day 25. Immunocytochemistry of TUBB3 was performed after in situ hybridization. **(F-K)** After the inhibition of FGF signaling with FGFR inhibitor PD 161570 during days 17–24, the number of RGC somas drastically reduced, and directional axon growth of RGCs was nearly absent, whereas FGF8 protein expression largely remained. CNTN2 immunocytochemistry before (F, H) and after FGF8 immunocytochemistry (G, I, J, K) are shown. N = 3/3 experiments. Scale bar, 250 µm (B), 100 µm (C-E), 1 mm (F), 200 µm (K).

Directional RGC axon growth toward and then along PAX2+ optic disc cells in CONCEPT organoids could be mediated by signaling molecules secreted from PAX2+ optic disc cells. scRNA-seq analysis identified expression signatures of cluster 2, the major component of PAX2+ optic disc cells (Fig. S10). Interestingly, FGF8 and FGF9 were differentially expressed in cluster 2 (Figs. 4K, 4L; 7A; S10). To assess the roles of FGF8 and FGF9 in CONCEPT organoids, we validated that FGF8 and FGF9 were expressed in PAX2+ optic disc cells of CONCEPT organoids (Fig. 7B-E). TUBB3+ RGC axons grew towards and then along the regions with high-level FGF8 and FGF9 (Fig. 7D, E), suggesting that FGF8 and FGF9 may attract RGC axon growth. FGFR1, FGFR2, FGFR3, MAP2K1, and MAP2K2 were expressed in multiple types of cells in CONCEPT organoids. In RGCs, FGFR1 and MAP2K2 were clearly expressed (Fig. S11), indicating that RGCs expressed the components that transduce FGF signaling. Since FGF8 and FGF9 are probably redundant and it is challenging to inactivate both FGF8 and FGF9 in CONCEPT organoids, we decided to inactivate FGF signaling with FGFR1/2/3 inhibitor PD 161570 during days 17–24, a stage at early RGC differentiation. After the FGFR inhibition, FGF8 expression was grossly unaffected. On the other hand, RGC somas were drastically reduced, and directional axon growth was nearly absent (Fig. 7F-7K). These findings indicate that a) PAX2+ optic disc cells differentially expressed FGF8 and FGF9; b) FGF signaling mediated by FGFRs is required for early RGC differentiation and directional axon growth in humans.

## Discussion

In this study, we report the self-formation of concentric zones of telencephalic and ocular tissues in CONCEPT telencephalon-eye organoids from human pluripotent stem cells, establishing a model for studying the early development of telencephalic and ocular tissues in humans. RGCs grew axons toward and then along a path defined by PAX2+ cell populations, setting up a model for studying RGC axon pathfinding. We identified expression signatures of cell clusters in CONCEPT organoids using single-cell RNA sequencing and revealed mechanisms of mechanisms of early RGC differentiation and axon growth. Lastly, we established a one-step method for the isolation of human electrophysiologically-excitable RGCs via CNTN2 under a native condition. Our studies not only provide deeper insight into coordinated specification of telencephalic and ocular tissues for directional RGC axon growth in humans, but also generate resources for therapeutic studies of RGC-related diseases such as glaucoma.

### The cyst is a radially symmetric epithelium mimicking the anterior ectoderm

Cysts are hollow spheres composed of homogeneous columnar epithelial cells with the apical surface at the lumen. They are induced from pluripotent stem cells via embedding small sheets of hESCs into Matrigel and subsequent growth either in a solid thin film ^47^ or suspensions ^28,33^. Cyst growth in suspensions is cost-effective and scalable. The formation of cysts induced by Matrigel mimics the epithelization of the epiblast by the extracellular matrix (ECM) in the blastocyst ^73,74^.

In this study, individual cysts efficiently generate telencephalic and ocular tissues in the absence of any extrinsic factors, indicating their default cell fates of the anterior ectoderm. In literature, anterior neural tissues are generated from re-aggregated single pluripotent stem cells through dual inhibition of Smad signaling ^75^ or inhibition of Wnt/ß-catenin signaling ^27^. Besides that, undirected cultures without extrinsic factors are used for the generation of retinal cultures ^29,30,76^. In our procedure, we did not add any extrinsic factors; the epithelial structure of cysts and subsequent adherent growth at a low density facilitate neural induction and differentiation. Our findings are consistent with the reports that epiblast cells are epigenetically primed for ectodermal fates ^77^ and neuroectodermal tissues are the default differentiation from pluripotent stem cells ^78,79^. Collectively, cysts mimic the anterior ectoderm in the epithelial structure and cell fates.

### Pattern formation and coordinated cell differentiation in CONCEPT telencephalon-eye organoids

Tissue patterning is fundamental for the formation of a body plan, which is defined by the anteroposterior, dorsoventral, and left-right axes. Self-formation of CONCEPT telencephalon-eye organoids establishes an efficient way to pattern early telencephalic and ocular tissues along an axis. In CONCEPT organoids on days 22–26, FOXG1+ telencephalon, PAX2+ optic stalk, PAX2+ optic disc, and VSX2+ neuroretinas are positioned along the center-periphery axis, mimicking the relative positions of those tissues in E10.5–13.5 mouse embryos. Our findings indicate that the formation of telencephalic and retinal tissues are highly coordinated, consistent with the prosomere model ^80,81^. Collectively, we establish CONCEPT telencephalon-eye organoids for studying the early formation of telencephalic and ocular tissues in humans.

The tissue patterning in CONCEPT telencephalon-eye organoids is originated from the attachment of a radially symmetric epithelium to the culture surface for growth as colonies since individual cysts kept in suspensions do not form any apparent tissue patterning. When a floating cyst initially contacted the culture surface, the contact resulted in ECM-cell adhesions. The initial ECM-cell adhesions caused additional ECM-cell contacts in neighboring cells, resulting in the flattening and spreading of a cyst onto the culture surface. Since the cyst is a radially symmetric epithelium, ECM-cell adhesions between the cyst and the culture surface formed sequentially in a concentric manner. Remodeling of cell-cell and ECM-cell adhesions and self-organization underlay the tissue patterning. Molecular events in concentric patterns during the attachment of a cyst onto the culture surface were eventually translated to concentric gradients of morphogens that specify cell fates. The radially symmetric epithelial structure of cysts is important for the formation of a concentric pattern since such pattern would not be generated when amorphous embryoid bodies are attached to the culture surface for growth as colonies. In literature, timed BMP4 treatment is shown to promote neuroretinal differentiation from pluripotent stem cells ^82^, and FGF8 promotes telencephalic and eye development ^5,22^. In our system, BMP4 and FGF8, along with BMP7 and other FGFs, were highly expressed starting at early stages and gradually formed concentric morphogen gradients, which would dictate tissue patterning, resulting in coordinated specification of telencephalic, optic stalk, optic disc, and neuroretinal tissues along the center-periphery axis in CONCEPT telencephalon-eye organoids. Co-culture of these tissues in the concentric zones provides mutual cues for coordinated tissue specification and growth. Nevertheless, anchorage culture of cell colonies prevents three-dimensional morphogenesis as seen in embryos, impeding proper tissue interactions at more advanced stages. When these cell colonies are detached using dispase for suspension culture, retinal progenitor cells self-organize to form protruding, translucent spheres called retinal organoids whereas other types of cells form structures with dark appearance; concentric zones no longer exist ^28^. It is conceivable that the simple suspension culture does not have proper physical and chemical supports provided by periocular tissues and other surrounding tissues in vivo and therefore optic stalk cells do not form a tube-like structure. It is a challenge ahead to reconstruct this three-dimensional environment to mimic the morphogenesis in vivo.

Concentric patterns of stem cell-derived cultures are also reported in a few other experiments. When dissociated single pluripotent stem cells are grown in micropatterned culture surface at certain cell densities in a medium supplemented with BMP4, concentric zones of progenitors expressing markers for trophectoderm, endoderm, mesoderm, and ectoderm are found ^43,44^. Cell density and colony geometry dictate cell fate specification from pluripotent stem cells. A concentric gradient of BMP4 activity regulates the patterning ^43^. When dissociated single pluripotent stem cells are grown in pre-patterned geometrically confined culture surface in a medium supplemented with dual inhibitors for TGF-β and BMP4, concentric zones of progenitors expressing the markers for neural plate and neural plate border are observed. Morphogenetic cues—cell shape and cytoskeletal contractile force—dictate the patterning of the neural plate and neural plate border via BMP-SMAD signaling ^45^. When dissociated single pluripotent stem cells are grown as individual colonies in a medium supplemented with knockout serum replacement, multiple zones of ectodermal cells autonomously form ^46^. In all three experiments, dissociated single cells are used to generate cell colonies through either cell re-aggregation or proliferation. In our case, adherent culture of a radially-symmetric epithelium – the cyst – was used to generate CONCEPT organoids. Among the three previous and our experiments, starting cells differ in their developmental potentials, tissue structures, and treatments. Despite the differences, all these organoids comprise concentric zones of distinct cell populations. Formation of CONCEPT telencephalon-eye organoids mimics the coordinated specification of early telencephalic and ocular tissues in humans.

### RGC axon pathfinding cues in CONCEPT telencephalon-eye organoids

In the mouse retina, multiple RGC axon guidance cues are concentrically organized around the optic disc, regulating RGC axon growth and exit from the eye through the optic stalk ^2^. Early differentiated RGCs are in a short distance from the nascent optic disc, and axons of later differentiated RGCs in more peripheral retinal regions follow the path of the initial axons. It is accepted that the optic-disc regions provide growth-promoting guidance cues whereas peripheral retinal regions provide inhibitory guidance cues ^2^. Pax2 is specifically expressed in the ventral optic stalk, optic vesicles, central neuroretina, optic disc, and optic stalk; Pax2 is essential for optic stalk and nerve development in mice ^15–17^.

In CONCEPT telencephalon-eye organoids, coordinated specification of telencephalic and ocular tissues generates PAX2+ optic-disc and optic-stalk cells that define the path for RGC axon growth. To the best of our knowledge, directional RGC axon growth guided by optic-disc and optic-stalk tissues has not been reported.

Single-cell RNA sequencing of CONCEPT organoids identified DEGs of PAX2+ optic-disc and optic-stalk cells. Interestingly, FGF8 and FGF9 were differentially expressed in PAX2+ optic-disc cells; inhibition of FGF signaling with an FGFR inhibitor during early RGC differentiation drastically decreased the number of RGC somas and nearly ablated directional axon growth, indicating that early RGC differentiation and directional axon growth require FGFR-mediated FGF signaling in humans. In chicks, Fgf8 and Fgf3 coordinate the initiation of retinal differentiation ^22,23^; FGF8 maintains Pax2 expression in optic nerve explants ^21^. In mice, FGF8 and FGF9 trigger axon outgrowth in motor neuron column explants ^83^. Therefore, the roles of FGF signaling in the regulation of early RGC differentiation and directional axon growth in CONCEPT organoids are consistent with literature. Dissecting DEGs of PAX2+ optic-disc cells in CONCEPT telencephalon-eye organoids elucidates the mechanisms of RGC axon growth and pathfinding in humans.

RGCs described in this study are still at their early stages in development. One prominent feature of these RGCs are the growth of their axons towards and long the path defined by optic disc cells, which is quite clear in CONCEPT organoids. Optic stalk cells in CONCEPT organoids confine RGC axon growth at inner regions, preventing RGC axons to enter telencephalic areas. We did not observe any clear evidence of synaptic or dendritic connections between RGCs and other cells at tested stages. Engineering assembloids may be needed to establish connections between RGCs and their visual targets.

### One-step isolation of developing human RGCs under a native condition

RGCs are degenerated in glaucoma, a major cause of vision impairment in developed countries. Disease modeling and drug discovery to suppress RGC death will have a huge impact on saving vision. The use of human RGC models is critical for therapeutic studies since humans and rodents differ significantly in RGCs. Additionally, cell replacement therapies for glaucoma are extensively evaluated. Therefore, efficient isolation of human RGCs in a native condition will have a substantial impact on therapeutic studies of RGC-related diseases such as glaucoma. In literature, cell surface marker Thy1 is used for the isolation of adult mouse RGCs, but it is unsuccessful in the isolation of RGCs from 3D retinal organoids ^84^. A study reports the purification of human RGCs via Thy1 from adherent cultures ^85^. Tagging human RGCs with an engineered marker Thy1.2 leads to efficient isolation of human RGCs ^84^, but may not be unsuitable for clinical uses. Using single-cell RNA sequencing, we identified cell surface protein CNTN2 as a specific marker for developing human RGCs. We isolated human electrophysiologically-excitable RGCs using MACS via CNTN2. Therefore, we establish a one-step method for isolating human RGCs under a native condition, facilitating therapeutic studies for RGC-related retinal diseases such as glaucoma.

## Materials and Methods

### Maintenance of hESCs

ESCRO and IRB committees at AECOM approved the use of hESCs in this project. Undifferentiated H1 hESCs (WiCell WA01) or hiPSCs (Corriell Institute AICS 0023) were grown on Matrigel-coated 6-well plates in mTeSR1 medium and passaged using ReLeSR (STEMCELL technologies) following manufacturer instructions.

### Retinal cell differentiation

CONCEPT telencephalon-eye organoids were generated as follows. A humidified incubator at 37°C with 5% CO2 was used for cell culture. H1 hESCs or iPSCs that were passaged using ReLeSR two or three days before experiments were detached using Dispase (GIBCO 17105041) and then harvested by centrifugation. After that, the cell pellets were suspended in ice-cold Matrigel. After gelling at 37 °C for 15–20 minutes, the hESC/Matrigel clump was gently dispersed in a N2B27 Medium (DMEM/F12+GlutaMAX (GIBCO):Neurobasal medium (GIBCO) = 1:1, 0.5 x B27 supplement (GIBCO), 0.5 x N2 supplement (GIBCO), 0.1 mM β-mercaptoethanol, and 0.2 mM L-GlutaMax) for floating culture. With the starting day of cell differentiation designated as day 0, cysts with a single lumen formed on day 1. Cysts with a single lumen constituted over 85% of cultures. On day 4 or 5, individual cysts with a diameter at around 150–200 μm were manually picked using a curved Pasteur pipets under an inverted microscope and then seeded onto Matrigel-coated 24-well plates at a density of 2–6 cysts per well. Cysts spontaneously attached to the culture surface and grew. From a time during days 13–16, attached cell colonies were grown in a KSR medium (GMEM medium supplemented with 10% knockout serum replacement, 1 mM sodium pyruvate, 0.1 mM non-essential amino acids, 2 mM l-glutamine, and 55 μM 2-mercaptoethanol; all the culture reagents were from Life Technologies). Culture mediums were changed every two or three days. Overall, the procedure is efficient and robust. Images in the manuscript represent 80–90% of cultures.

### Inhibition of FGF signaling in CONCEPT telencephalon-eye organoids with FGFR inhibitor PD 161570

To inactivate FGF signaling in CONCEPT organoids, FGFR1/2/3 inhibitor PD 161570 (1 µM; Tocris) was supplemented to the culture medium staring on day 17, with vehicle DMSO as a control. Treated CONCEPT organoids were harvested on day 24 for assays.

### Magnetic-activated cell sorting (MACS) of developing human RGCs

Retinal organoids in suspension culture were generated using the established method ^28,33^. For each MACS experiment, 84 – 140 retinal organoids at stages of day 41 – 70 were dissociated into single cells using Accutase (GIBCO A1110501). Non-retinal cells were trimmed if there were any. Dissociated single cells were harvested using centrifugation and then incubated for 25 minutes at room temperature with MagnaBind goat anti-mouse IgG (ThermoScientific 21354) beads that were previously coupled with a CNTN2 antibody (DSHB 4D7) following manufacturer instructions. Cells bound to the beads were isolated using a magnetic rack and then washed one time with the KSR medium supplemented with antibiotic:Antimycotic (GEMINI 400101) while the tube was still against the magnetic rack. After the wash, the cells were released from the beads via Accutase digestion for 30 minutes and then harvested using centrifugation. The isolated cells were plated onto a chamber slide (ibidi 80826, ibidiTreat µ-Slide 8 Well, coated with poly-ornithine and Matrigel, 30,000–50,000 cells/200 µl/well) in BrainPhys neuronal medium (Stem Cell Technology 05790) supplemented with N2 and B27 (GIBCO 17502001, A3582801). From 100 retinal organoids on day 41 to 48, around 385,000 RGCs were isolated. After 10 days of culture in chamber slides, RGCs were fixed in 4% paraformaldehyde (PFA) for 10 minutes and then processed for immunostaining.

### Immunostaining, antibodies, and light microscopy

CONCEPT telencephalon-eye organoids were fixed in 4% PFA for 15–30 minutes at room temperature and processed for immunostaining. See Supplemental Information for details.

### In situ hybridization

DIG-labeled anti-sense RNA probes for in situ hybridization were generated via in vitro transcription using a DIG RNA labeling kit (Millipore Sigma-Aldrich 11175025910). See Supplemental Information for details. To assess the in vivo expression of DEGs identified by single-cell RNA sequencing, in situ hybridization images of their mouse orthologs in E14.5 mouse brains were downloaded from a public database ^86^ (https://gp3.mpg.de/) with permission and then assembled in supplemental Figs.

### Electron microscopy (EM)

EM was performed by Analytical Imaging Facility in Albert Einstein College of Medicine with a standard method. Lens cell clusters were fixed in 0.1M Cacodylate buffer containing 2% paraformaldehyde and 2.5% glutaraldehyde for 60 minutes at room temperature and then processed for EM.

### Single-cell RNA sequencing

CONCEPT telencephalon-eye organoids at day 24 from one culture well were dissociated into single cells using activated Papain (Worthington Biochemical Corporation LS003126) following manufacturer’s instructions. Then, 10,000 dissociated cells were captured using Chromium Controller (10x Genomics), followed by library preparation using Single Cell 3’ version 3.1 kit (10x Genomics) following manufacturer’s instructions. The library was sequenced in one lane of Illumina HiSeq (2×150 bp) in GeneWIZ company.

Fastq sequences were mapped to the human genome (GRCh38–3.0.0) using CellRanger (3.1.0) to generate a count matrix, which was then analyzed using the Seurat Package (v3.2.0) ^59^. Sequenced cells were filtered (nFeature_RNA > 200 & nFeature_RNA < 6000 & percent.mt < 20), resulting in 10218 cells with high quality. Dimension reduction and clustering were performed using the following functions: *NormalizeData, FindVariableFeatures, ScaleData, RunPCA, ElbowPlot, FindNeighbors (dims = 1:17),* and *FindClusters (resolution = 0.5).* Differentially expressed genes were identified using the function *FindAllMarkers (only.pos = FALSE, min.pct = 0.25, logfc.threshold = 0.25)*.

### Whole-cell patch clamp recordings of isolated RGCs in culture.

Standdard methods were used. See Supplemental Information for details.

### Statistical analysis

Statistical analysis in DEG identification was performed using the Seurat Package (v3.2.0) ^59^. Adjusted p-values were shown.

## Supplementary Material

Supplement 1

## Figures and Tables

**Tab.1.** Cell counts of clusters in the scRNA-seq dataset

Cluster	0	1	2	3	4	5	6	7	8	9	10	11	12	13
Idents	Tel/R	Tel	OD	Tel	Tel/OS	NR	RPE	NR	Tel/OS	Tel/OS	Tel	RGC	Tel	UD
# Cells	1295	1158	964	931	921	871	747	743	729	603	450	393	333	80

Abbreviations: Idents, assigned cell identities; # Cells, cell number; Tel, telencephalon; R, retina; OD, optic disc; OS, optic stalk; RPE, retina pigment epithelium; RRG, retinal ganglion cell; UD, undetermined.
