# Supplementary material for: Self-formation of concentric zones of telencephalic and ocular tissues and directional retinal ganglion cell axons
Source: bioRxiv. 2023 Mar 24:2023.03.22.533827. Preprint. [Version 1] doi: 10.1101/2023.03.22.533827 (PMC10055356; doi:10.1101/2023.03.22.533827)
Supplement: Supplement 1 [file NIHPP2023.03.22.533827v1-supplement-1.pdf]

bioRxiv preprint doi: https://doi.org/10.1101/2023.03.22.533827; this version posted March 24, 2023. The copyright holder for this preprint (which was not certified by peer review) is the author/funder, who has granted bioRxiv a license to display the preprint in perpetuity. It is made available under aCC-BY 4.0 International license.

Supplemental Information

Immunostaining, antibodies, and light microscopy

CONCEPT telencephalon-eye organoids were fixed in 4% PFA for 15-30 minutes at room temperature and processed for immunostaining. These primary antibodies were used: FOXG1 (Abcam, ab18259, 1:500), TUBB3 (Covance MMS-435P, 1:1000), FGF8 (1:500, R&D MAB323), RBPMS (1:200, PhosphoSolution 1830-RBPMS), ISL1 (1:500, DSHB 40.2D6), SNCG (1:200, Abcam ab55424), PAX2 (Invitrogen, 716000, 1:200), alpha A crystallin (Santa Cruz sc 22743, 1:500, shown as CRYAA in figure panels), beta crystallin (Santa Cruz sc-22745, 1:100, shown as CRY B in figure panels), CNTN2 (DSHB 4D7, 1:100), ALDH1A3 (Invitrogen, PA529188, 1:500), VAX1/2 (Santa Cruz sc-98613, 1:200), PAX6 (1:500, Covance PRB-278P), POU4F2 (Santa Cruz, SC-6026, 1:200), SIX3 (1:500, Rockland), and VSX2 (1:500, Millipore AB9016). Primary antibodies were visualized using Alexa Fluor 488-, 568-, and 647-conjugated secondary antibodies and imaged using a Zeiss AxioObserver Z1 microscope. When the sample did not fit in one image, multiple images were stitched to obtain an overview. In dual-color immunocytochemistry for PAX2 and FGF8, FGF8 was visualized using AP-conjugated anti mouse secondary antibody (Invitrogen A16038), and PAX2 was detected by biotin-conjugated anti rabbit secondary antibody (Invitrogen B2770) followed by HRP-conjugated streptavidin (ThermoScientific 21130). For dual color immunocytochemistry of CNTN2 and FGF8 (both are mouse antibodies; no working antibodies raised in different species are available), immunocytochemistry of CNTN2 was performed first and visualized using HRP-conjugated streptavidin. Then, the samples were fixed in 4% PFA for 15 minutes to avoid antibody crosstalk. After that, FGF8 antibody was applied and then detected by an AP-conjugated secondary antibody. Brown and blue colors are well separated in Fig. 5K, indicating that antibody crosstalks did not exist.

bioRxiv preprint doi: https://doi.org/10.1101/2023.03.22.533827; this version posted March 24, 2023. The copyright holder for this preprint (which was not certified by peer review) is the author/funder, who has granted bioRxiv a license to display the preprint in perpetuity. It is made available under aCC-BY 4.0 International license.

PCR Primers for in situ hybridization probes

PCR primers are as follows: BMP4: forward, CGGAAGCTAGGTGAGTGTGG, reverse, GAGtaatacgactcactatagggGGAAGCCCCTTTCCCAATCA; BMP7: forward, gaggtccctctccattccct, reverse, GAGtaatacgactcactatagggtgcacccatcagacctccta; FGF8: forward, GTTGCACTTGCTGGTCCTCT, reverse, GAGtaatacgactcactatagggTTGAGTTTTGGGTGCCCTAC; PAX2: forward, gctgtctgtgctgtgagagt, reverse, GAGtaatacgactcactatagggccggggacatttagcaggtt; SEMA5A: forward, CAGAGGCTCAGGCACAATGA, reverse, GAGtaatacgactcactatagggTCCGTGTCTACCCAGGACTT; CYP1B1: forward, cccagcggttcttcatgagt, reverse, GAGtaatacgactcactataggggcacacttggttgcgttagt; LEFTY2: forward, agccctctaactgaacgtgtg, reverse, GAGtaatacgactcactatagggtcttctgagtatctacattcaattgct; EMX2: forward, ACCGAGAAAGGGAGAGGGAA, reverse, GAGtaatacgactcactatagggTCGGCCAATTTCTCCAACCA; FGF9: forward, GTCCGCTATGAACCTGTGGT, reverse, GAGtaatacgactcactatagggATAGTCTCGCTTGCCCAAGG; FGF2: forward, aacaccgaaatgctggaggt, reverse, GAGtaatacgactcactataggggagaccacatgtacacgcca.

Whole-cell patch clamp recordings of isolated RGCs in culture.

RGCs were isolated from retinal organoids on day 48 using MACS via a CNTN2 antibody and then grown on polymer coverslips in chamber slides (ibidi 80826) for 20 – 27 days. At the time of whole-cell patch clamping recordings, the polymer coverslips carrying RGCs were carefully cut out with a scalpel blade and then placed in a recording chamber under an upright microscope (Zeiss Examiner A1), containing artificial cerebrospinal fluid (aCSF) composed of (*in mM*): NaCl (*140*), $MgCl_2$ (*1*), KCl (*5*), $CaCl_2$ (*2*), Hepes (*10*), Glucose (*10*). Osmolarity and pH were adjusted to 300mOsm and 7.3 respectively. Whole-cell patch-clamp recordings in voltage and current-clamp mode were obtained at room temperature using an Optopatch amplifier

bioRxiv preprint doi: https://doi.org/10.1101/2023.03.22.533827; this version posted March 24, 2023. The copyright holder for this preprint (which was not certified by peer review) is the author/funder, who has granted bioRxiv a license to display the preprint in perpetuity. It is made available under aCC-BY 4.0 International license.

(Cairn Research, UK) and acquired with WinWCP 5.2 freeware (John Dempster, SIPBS, University of Strathclyde, UK). Patch pipettes (3-4MΩ when filled with corresponding solution) were pulled from borosilicate capillaries using a horizontal puller (Sutter P97, USA) and coated with dental wax to reduce the pipette capacitance. For current clamp recordings, patch pipettes were filled with a solution containing (*in mM*): K-gluconate (*130*), Na-gluconate (*10*), NaCl (*4*), Hepes (*10*), Phosphocreatin (*10*), MgATP (*4*), Na_2GTP (*0.3*). Osmolarity and pH were adjusted at 295mOsm and 7.3 respectively. Resting membrane potential was obtained from averaging membrane potential recorded for one minute in I=0 mode immediately after breaking the cell membrane. In current clamp mode, with the cell hyperpolarized to -70 mV, current steps of 100 to 500pA were made to explore whether the cells were excitable. For voltage-clamp recordings, cells were held at clamped potential of -80mV, and series resistance was monitored and compensated (>80%). Membrane potentials were corrected for the liquid junction potential, calculated at +5.4mV (https://swharden.com/LJPcalc). Patch pipettes were filled with a solution containing (*in mM*): KCl (*140*), $MgCl_2$ (*5*), $CaCl_2$ (*2.5*), Hepes (*10*), MgATP (*4*), Na_2GTP (*0.3*). Osmolarity and pH were adjusted at 295mOsm and 7.3 respectively. A -P/4 subtraction protocol was used to isolate voltage-gated currents by removing linear leak current and capacitance artifacts. In all cases, voltage and current recordings were low pass filtered at 3kHz and digitized at 10-20kHz (Axon Digidata 1550b, Molecular Device, USA). Tetraethylammonium-chloride (TEA, Sigma-Aldrich, USA) and Tetrodotoxin-citrate (TTX, Fisher Scientific) stocks were diluted in aCSF to reach 20mM and 1μM respectively. All recordings were analyzed with WinWcp 5.2 and custom scripts and routines written in Python 3.9. Statistical tests were performed with corresponding function from *scipy.stats* package (v1.8).

bioRxiv preprint doi: https://doi.org/10.1101/2023.03.22.533827; this version posted March 24, 2023. The copyright holder for this preprint (which was not certified by peer review) is the author/funder, who has granted bioRxiv a license to display the preprint in perpetuity. It is made available under aCC-BY 4.0 International license.

Legends for supplemental Figures

Fig. S1. Reproducibility of CONCEPT telencephalon-eye organoids is demonstrated by consistent gene expression profiles of multiple organoids in whole culture wells. Related to Figs. 1, 7. **(A-C)** CONCEPT organoids on day 17 expressed FGF8 (A, n =5/5), BMP4 (B, n = 4/4; the broken colony was not counted since its morphology is not quite clear), and BMP7 (C, n = 4/4) in concentric zones. **(D-F)** CONCEPT organoids on day 25 expressed FGF8 (D, n = 5/8), RGC marker CNTN2 (E, n = 5/6), and telencephalon marker EMX2 (F, n = 2/2) in concentric zones. Scale bars, 1 mm (A-F).

Fig. S2. Mesoderm, endoderm, and neural crest markers are not expressed in CONCEPT telencephalon-eye organoids at 24. Related to Fig. 4. The dataset for Fig. 4 is used for plotting. Expression profiles of mesoderm markers TBXT, GATA2, and HAND1, endoderm markers GATA1, GATA4, and SOX17, neural crest markers SNAI1, SOX10, FOXD3 are shown.

Fig. S3. The expression of top DEGs in cluster 0. Related to Fig. 4. The dataset for Fig. 4 is used for plotting. Cluster 0 was marked by DEGs that were lowly expressed.

Fig. S4. Diencephalon markers and midbrain/hindbrain markers are rarely expressed in CONCEPT telencephalon-eye organoids at day 24. Related to Fig. 4. **(A-F)** Diencephalon markers GBX2, WNT3, and SOX14, and midbrain/hindbrain markers EN2, PAX7, and TFAP2B were rarely expressed. The dataset for Fig. 4 is used for plotting. **(G-L)** Expression of the diencephalon markers and midbrain/hindbrain markers in the E14.5 mouse brain. The images were downloaded from a public database (https://gp3.mpg.de/) with permission. Image IDs were shown following gene symbols.

Fig. S5. DEGs for telencephalic clusters in CONCEPT telencephalon-eye organoids include markers for both the pallium and subpallium. Related to Fig. 4 and Fig. S6. DEGs

bioRxiv preprint doi: https://doi.org/10.1101/2023.03.22.533827; this version posted March 24, 2023. The copyright holder for this preprint (which was not certified by peer review) is the author/funder, who has granted bioRxiv a license to display the preprint in perpetuity. It is made available under aCC-BY 4.0 International license.

for telencephalic clusters were used for plotting, and expression of their orthologs in E14.5 mouse brain was shown in Fig. S6. **(A-D)** Expression of the DEGs POU3F3, RGMA, EDNRB, and SOX13, which orthologs are expressed in the pallium of E14.5 mouse brain (see also Fig. S6B-E). **(E-L)** Expression of the DEGs DLX2, RGS16, DLX1, DLL1, DLX6-AS1, NEFL, DCX, and RTN1, which orthologs are expressed in the subpallium of E14.5 mouse brain (see Fig. S6F-M).

Fig. S6. Mouse orthologs of the telencephalic DEGs are expressed in both the pallium and subpallium of the E14.5 mouse brain. Related to Fig. 4 and Fig. S5. The images were downloaded from a public database (https://gp3.mpg.de/) with permission. Image IDs were shown following gene symbols. **(A)** Expression of Foxg1. **(B-E)** Expression of the genes Pou3f3, Rgma, Ednrb, and Sox3 in the pallium of E14.5 mouse brain. **(F-M)** Expression of the genes Dlx2, Rgs16, Dlx1, Dll1, Dlx6os1, Nefl, Dcx, and Rtn1 in the subpallium of E14.5 mouse brain.

Fig. S7. DEGs in cluster 6 include RPE markers. Related to Fig. 4. Top DEGs in cluster 6 include RPE markers PMEL, HSD17B2, DCT, and MITF.

Fig. S8. Mouse orthologs of the markers for Pax2+ optic disc and Pax2+ optic stalk cell clusters in CONCEPT organoids are indeed expressed in the optic disc and optic stalk/nerve, respectively, in E14.5 mouse embryos. Related to Fig. 4. In situ hybridization images of the E14.5 mouse brain were downloaded from a public database (https://gp3.mpg.de/) with permission. Image IDs were shown following gene symbols. **(A-T)** Expression of the gene markers in E14.5 brain is shown.

Fig. S9. CONCEPT telencephalon-eye organoids are generated using hiPSC line AICS 0023. Related to Fig. 7. hiPSCs (AICS 0023) were used for the generation of CONCEPT

bioRxiv preprint doi: https://doi.org/10.1101/2023.03.22.533827; this version posted March 24, 2023. The copyright holder for this preprint (which was not certified by peer review) is the author/funder, who has granted bioRxiv a license to display the preprint in perpetuity. It is made available under aCC-BY 4.0 International license.

telencephalon-eye organoids. **(A-D)** POU4F2+ RGCs grew CNTN2+ axons toward and then along a circular path defined by PAX2+ cell populations. Scale bar, 500 μm (A), 100 μm (B).

Fig. S10. Expression signatures of cell cluster 2. Related to Figs. 4, 5. **(A)** A list of top 10 DEGs and additional DEG markers SEMA5A, PAX2, COL13A1, FGF9, and FGF8 in cluster 2 were identified using Seurat analysis. **(B-E)** Expression of PAX2, SEMA5A, CYP1B1, and LEFTY2 in CONCEPT organoids at day 25 was revealed by in situ hybridization. The PAX2+ optic disc at the outer zone was marked (bracket in B).

Fig. S11. FGFR1, FGFR2, FGFR3, MAP2K1, and MAP2K2 are expressed in multiple types of cells in CONCEPT organoids. Related to Fig. 4. In RGCs, FGFR1 and MAP2K2 were expressed.
